# Chitosan and Whey Protein Bio-Inks for 3D and 4D Printing Applications with Particular Focus on Food Industry

**DOI:** 10.3390/molecules27010173

**Published:** 2021-12-28

**Authors:** Wei Yang, Anqianyi Tu, Yuchen Ma, Zhanming Li, Jie Xu, Min Lin, Kailong Zhang, Linzhi Jing, Caili Fu, Yang Jiao, Lingyi Huang

**Affiliations:** 1 Quality and Technology Center, Hainan Xiangtai Fishery Co., Ltd., Chengmai 571924, China; yw@esfish.com; 2 Fujian Key Laboratory of Inspection and Quarantine Technology Research, Fuzhou 350309, China; 3 Food Science and Technology Department, National University of Singapore (Suzhou) Research Institute, Suzhou 215123, China; a23tu@uwaterloo.ca (A.T.); yuchen.ma@nusri.cn (Y.M.); lizhanming@just.edu.cn (Z.L.); jie.xu@nusri.cn (J.X.); linzhi.jing@nusri.cn (L.J.); caili.fu@nusri.cn (C.F.); 4 Faculty of Science, University of Waterloo, Waterloo, ON N2L 3G1, Canada; 5 School of Pharmacy, Fujian Medical University, Fuzhou 350122, China; minlin_000@foxmail.com; 6 The Marketing Department, Beijing Zhongwei Research Center of Biological and Translational Medicine, Beijing 100071, China; zhangkl1124@163.com; 7 College of Food Science and Technology, Shanghai Ocean University, Shanghai 201306, China

**Keywords:** 3D printing, 4D printing, chitosan, whey protein, cultured meat

## Abstract

The application of chitosan (CS) and whey protein (WP) alone or in combination in 3D/4D printing has been well considered in previous studies. Although several excellent reviews on additive manufacturing discussed the properties and biomedical applications of CS and WP, there is a lack of a systemic review about CS and WP bio-inks for 3D/4D printing applications. Easily modified bio-ink with optimal printability is a key for additive manufacturing. CS, WP, and WP–CS complex hydrogel possess great potential in making bio-ink that can be broadly used for future 3D/4D printing, because CS is a functional polysaccharide with good biodegradability, biocompatibility, non-immunogenicity, and non-carcinogenicity, while CS–WP complex hydrogel has better printability and drug-delivery effectivity than WP hydrogel. The review summarizes the current advances of bio-ink preparation employing CS and/or WP to satisfy the requirements of 3D/4D printing and post-treatment of materials. The applications of CS/WP bio-ink mainly focus on 3D food printing with a few applications in cosmetics. The review also highlights the trends of CS/WP bio-inks as potential candidates in 4D printing. Some promising strategies for developing novel bio-inks based on CS and/or WP are introduced, aiming to provide new insights into the value-added development and commercial CS and WP utilization.

## 1. Introduction

As reported in previous research, multi-material 3D printing technology (Fab@Home) has been employed to print chocolate, cheese, biscuits, etc. [1]. Three-dimensional printing starts with a build-up of a model by using computer-aided design (CAD) software, which converts physical characteristics of products into computational data [2]. In addition, the model is sliced horizontally into layers of images and sent to a 3D printing machine for layer-by-layer manufacturing. Depending on the 3D printing technique selected, a single layer thickness of the construct varies between microns and millimeters. To accomplish the printing of an object, a layer-by-layer superposition must be applied. This means that the printer repeatedly constructs a new thin layer of material based on the previously printed layer, according to the sliced images of a 3D object [2,3]. As the extension of 3D printing, the products of 4D printing are able to change their properties or functionalities predictably in response to external stimuli, such as time, electric, temperature, humidity, etc. [3]. Four-dimensional printing originated from 3D printing, adding the fourth dimension of “time”, but in contrast to 3D printing, only stimulable smart materials can be used for 4D printing. For example, Chen et al. used UV irradiation of ergosterol-incorporated purple-sweet-potato paste to achieve nutritional change [4]. Compared with 3D printing technology, 4D printing presents the advantage of dynamic adaptability and the ability to print more refined structures. This technology is a breakthrough in the field of biomedical engineering, solving the limitations of 3D printing and comprehensively advancing the process of bioprinting. The shape-memory materials (SMM) were developed in the early 2000s and directly incorporate into the deformation and reformation of 4D-printed products on the basis of multilayer manufacturing [5,6]. Currently, multiple 3D printing technologies have been applied in many sectors, such as fused deposition modeling (FDM, or fused filament manufacturing), stereolithography (SLA), electro-hydraulic printing (EHDP), selective laser sintering (SLS), etc. These techniques are innovated to adapt different characteristics of printing materials [7]. Materials are divided into three categories: solid-based, liquid-based, and powder-based. The materials’ utilization, advantages, and disadvantages of FDM, SLA, EHDP, SLS, and Selective Laser Melting (SLM), as well as the 4D printing technologies, are analyzed in Table 1.

**Figure 1:**
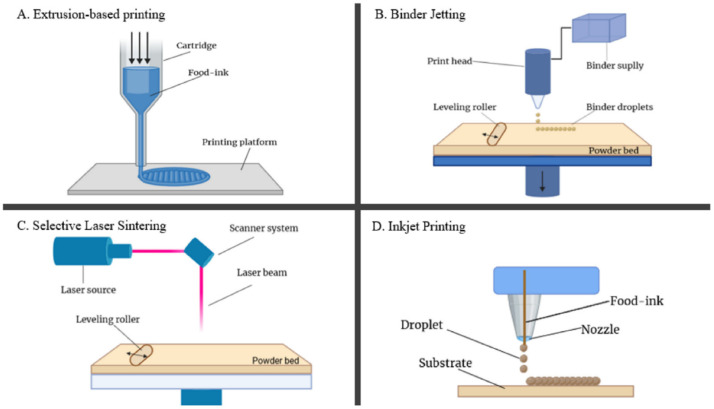
Detailed working diagram of four types of 3D printing technologies: (**A**) extrusion-based printing, (**B**) binder jetting, (**C**) selective laser sintering, and (**D**) inkjet printing. Adapted from Reference [15], with permission from Elsevier.

Three-dimensional printing has great potential in the food sector because of the economic and environmental benefits of the technology, the ability to create complex structures, and the applicability large-volume industrial production. It allows for the creation of privately customized food products according to nutrient demands, as well as unique textures, colors, and tastes. There are four types of 3D printing technologies utilized in the food industry: extrusion-based printing, inkjet printing, binder jetting, and selective laser sintering (Figure 1) [15]. In addition, FDM, as mentioned above, is the most used technique in the food industry, and it usually utilizes hummus, chocolate, and sweets as printing inks [16].

As the extension of 3D printing, the products of 4D printing are able to change their properties or functionalities predictably in response to external stimuli, such as time, electric, temperature, humidity, etc. [3]. The shape-memory materials (SMMs) were developed in the early 2000s and directly incorporate to the deformation and reformation of 4D-printed products on the basis of multilayer manufacturing [5,6], as well as shape-memory polymers (SMPs), shape-memory polymer composites (SMPCs), shape-memory alloys (SMAs), and shape-memory hybrid materials (SMHs). However, not only 3D printing but also 4D printing technologies have their advantages and disadvantages in practical applications in the medical field or the food sector (Table 2)

No matter if it is 3D or 4D, matching materials and technologies are essential for successful printing. At present, many food-derived materials have been developed as inks [18]. Among them, CS and WP are potential bio-ink candidates. CS is the polysaccharide produced by N-deacetylation of chitin, which is found on the shell of shrimps and other crustaceans [19], usually as the waste by-product in seafood industries [20]. The deviates of CS have a variety of advantages in medicine as a drug-deliverer [21], in agriculture as antimicrobial agents and biopesticides [22], in clinical applications for hemostasis and bacterial control [23], and in food industries for food packaging [24].

CS has excellent biocompatibility, biodegradability, antibacterial activity, non-carcinogenicity, and non-immunogenicity, and it was used mainly in the research of scaffold construction for the cell growth of animal tissues and organs [25]. Recently, the microstructured chitosan-based hydrogel was investigated to improve cell growth, regeneration, and cell adhesion, which provided positive and consistent effects to tissue engineering [26,27,28]. Therefore, in the process of producing cultured meat by 3D printing, CS served as a printing material for scaffolds construction by taking advantage of the edibility and antibacterial properties [29,30] and then uniform living cells or cell populations. It is distributed on the surface of the preprinted 3D scaffold, allowing cells to attach, fuse, and grow according to the structure of the scaffold [31].

There are various types of protein in whey, such as α-lactalbumin, β-lactoglobulin, glycomacropeptide, serum albumins, immunoglobins, etc. [32]. These proteins are claimed to support muscle growth, development, and recovery. Overall, the special physical properties, chemical properties, and nutritive value make WP an important ingredient in the food industry [33]. WPs are also facilitated widely in emulsion, gelation, and thickness [34] and used as additives to improve the printability, stability, and performance of bio-inks for the food-grade 3D printing products.

Both CS and WP are well utilized as ideal materials with excellent biochemical, structural, and antioxidant properties [35]. Numerous reports have been presented on the applications of CS and WP recently. Even though there are reviews that analyze the biomedical properties and processing performances of CS and WP during 3D printing, a systematic review on utilizing CS and WP as bio-inks for additive manufacturing is still needed. This review summarizes the preparation of CS and/or WP as bio-ink that fulfill the limitations, requirements of 3D/4D printing, and post-treatment of printed objectives in the food sector. Moreover, the applications of CS and WP in 3D printing were also introduced in this review, providing new insights for further utilization in food 3D/4D-printing fields.

## 2. Chitosan in 3D and 4D Printing

CS has widely used in food preservation and vegetable or fruit coatings, due to its antimicrobial and antioxidative properties. In addition, the US Food and Drug Administration (FDA) also approved CS as an oral dietary fiber and food additive [36,37]. The printability, biodegradability, strength, and flow of CS greatly depend on the molecular weight and angle of the acetylation process. In previous studies, CS alone might not be considered as a good bio-ink, and the hydrophobic properties and shrinkage in size lead to low precise geometries and low stability of the final products [38]. However, various research studies have suggested that the combination of CS and other substances, such as PLA, gelatin, and silk proteins, would significantly improve the printability and stability of CS [39].

### 2.1. Chitosan-Related Nanotechnologies in 3D Printing

**Figure 2:**
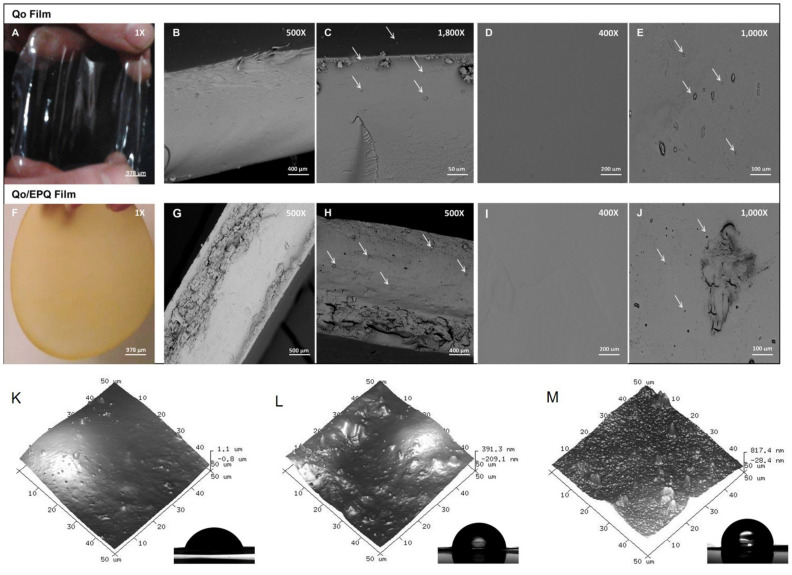
Camera pictures and the Scanning Electron Microscope pictures of NQoThs in Qo and Qo/EPQ films. (**A**) Camera picture of Qo film. (**B**,**C**) Cross-section of Qo film. (**D**,**E**) Surface of Qo film. (**F**) Camera picture of Qo/EPQ film. (**G**,**H**) Cross-section of Qo/EPQ film. (**I**,**J**) Surface of Qo/EPQ film. (**K**) Surface of Tara gum film. (**L**) Surface of Tara gum film incorporated with bulk CS. (**M**) Surface of Tara gum film incorporated with CS nanoparticles. Adapted from References [42,46], with permission from Elsevier.

Based on an environmental protection concept, the search for green alternatives to plastics has become a focus of attention. In the field of biological packaging films, CS has become the most suitable natural biomaterial for the manufacture of bio-composites, due to its non-toxicity, low cost, and biodegradability [40]. Various studies have considered that CS products can significantly improve food freshness and reduce the cost and waste during storage; moreover, CS packaging has been introduced for the storage of fresh foods [41]. A chitosan–protein hybrid film was developed with higher flexibility and elongation properties [42,43]. Meanwhile, applying nanoparticles in the film can restraints the diffusion of gases and water vapors [44,45]. Caro et al. evaluated the antimicrobial activity and 3D-film-printing properties of CS with quinoa protein extract that was loaded with nanoparticles and concluded enhanced bacteria growth inhibition, water, and gas permeabilities, resulting in greater efficiency of delivering active compounds than the control CS film [32] (Figure 2A–J). In addition, research on edible Tara gum containing CS nanoparticle films revealed a rough hydrophobic surface (Figure 2 K–M), which had better thermal stability with lower water vapor permeability and solubility, while bulk CS films exhibited higher antimicrobial properties [46].

Three-dimensional printing plays a key role in the preparation of composite materials because of its ability to generate complex structures and to verify the results of composite materials quickly. Liu et al. combined CS to inhibit the growth and reproduction of microorganisms, using the tubular structure of halloysite nanotubes (HNTs) to achieve the encapsulation effect and the antioxidant activity of tea polyphenols (TP) and prepared CS/HNTs-TP composite material. The results show that the composite material has a significant effect on the preservation of blueberries [47].

Three-dimensional printing has significant advantages in customizing the dosage and the release profile of new drugs. Thus, this technology has a wide range of applications in protein/peptide controlled-release therapy or tissue regeneration. Wang et al. developed a 3D printing with complex shapes and structures by wrapping the protein in the CS nanogel and incorporating it into the mixed suspension. The results show that the molecular weight of the CS prepared from the nanoparticle is directly proportional to the release rate of the composite structure [48]. This methodology can be used to achieve sustained release of macromolecular proteins to guide tissue growth and maintain alignment in the cultured meat. Incorporating 3D printing and drug release conforms to the research direction of cultured meat, shows good biocompatibility, and has a wide application prospect. The recent uses of 3D printing in lab-grown meat are discussed in the next section.

### 2.2. Manufacture of Cultured Meat

Cultured meat is a hot field that many researchers have studied in recent years, with broad prospects for development. The industrialized large-scale production of cultured meat is based on the value proposition of sustainability, which not only reduces labor costs, energy consumption, and air pollution, but also facilitates the establishment of a sustainable supply chain, reduces waste and loss, and promotes consumption. It is necessary to use the five different stages of the innovative decision-making process to increase consumer acceptance.

**Figure 3:**
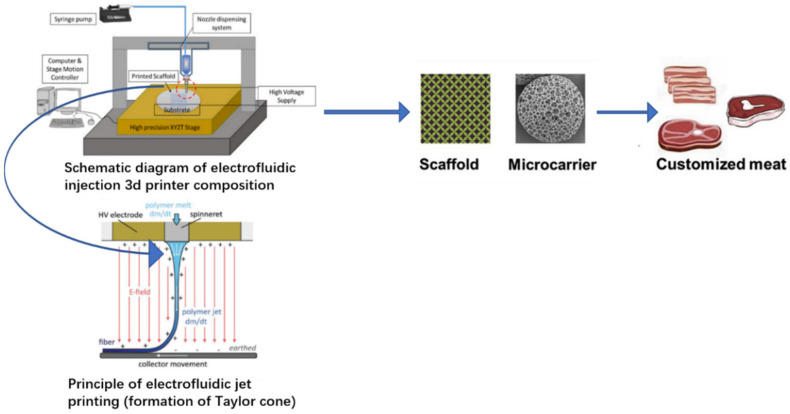
Three-dimensional printing process of cultured meat. Reprinted with permission from Reference [50] from American Chemical Society. Adapted from Reference [51], with permission from Elsevier.

The key to the development of cultured meat is to reshape the meat-like texture, so different 3D printing methods are derived based on the four types of printing materials used. One of the printing materials is biomaterials: arranging muscle cells in a single axis and creating the structure for further functions. This is similar to natural muscle tissue (Figure 3) [49].

Currently, cultured meat is classified into three major categories: scaffold construction, starter cell selection, and processing and incubation methodology. Among them, 3D printing contributed to the scaffold construction by providing a refined and multilayer structure that allows different tissues and cells to grow rapidly. The cultured meat scaffold must be removable after tissue formation of edible and heat-resisting materials [49]. 

Three-dimensional printing technology allows the preparation of high-precision scaffolds because of a cryogenic platform to ensure biological activity. CS shows great potential in the field of tissue cell scaffolds, due to its good biocompatibility and broad-spectrum antimicrobial effect. Moreover, 3D-printed CS can be prepared into composites to improve degradation properties and mechanical properties. Park et al. prepared composites with methyl cellulose (CMC), which has a good affinity with CS and induced chemical crosslinking between them, which increased the total porosity of the films and was more favorable for cell growth. Moreover, the introduction of crosslinking made the blue microalgae intermediate active ingredient C-phycocyanin (CPC) bind and electrostatically interact with polysaccharides, which ensured the stability of CPC. Moreover, CPC was used as a serum replacement nutrient, significantly reducing the production cost of cultured meat. From the above, it can be concluded that CS significantly enhances the biological properties of scaffolds while reducing the cost; thus CS can be considered a wise choice of material, as it is capable of achieving the biological function of 3D-printed scaffolds, with a wide range of future applications in the field of cultured meat [52].

### 2.3. Polyelectrolyte Complex Gels in 3D Printing

Polysaccharides, such as κ-carrageenan, alginate, pectin, gelatin, and CS, are widely exists in natural resources. These polysaccharides have various bioactivities [53,54] and good physicochemical properties [55,56], which are suitable for the production of hydrogels. According to the chemical properties, the hydrogels are characterized into two subcategories, anionic and cationic. Modifications and mixtures of polysaccharides or hydrogels are suggested to meet the requirements of different bio-utilities. Hence, Kean’s study found that the combination of anionic and cationic hydrogels formed polyelectrolyte complex gels, providing great potential 3D bio-printing properties. CS and carrageenan formed a polyelectrolyte complex gel [57], while the polyelectrolyte hydrogels have excellent performance in the 3D printing of soft actuators and robots [58]. Moreover, the nanocoated polyelectrolyte gel of CS and pectin was applied in fruit preservation as the outer membrane [59]. The presence of the coating can reduce oxidation and discoloration and can also delay the development of microorganisms to extend their shelf life [41]. Therefore, the CS and carrageenan polyelectrolyte gel are potential materials in 3D printing of food preserving membrane, and further studies are suggested to perform for the discovery of the properties and functionalities.

### 2.4. Hydroxybutyl Methacrylated Chitosan in 4D Printing

As an extension of 3D printing, products of 4D printing can change their physical properties in response to external or internal stimuli with time [60]. Exploring more food-grade materials in 4D printing is becoming a popular research target in food sector. Compared with 3D printing, 4D printing of personalized and healthy food still requires a large amount of research to satisfy the commercial markets. As mentioned above, the SMM can memorize and reform the original shape after deformation in response to the external stimuli, which are considered as 4D printing materials. According to the review by Teng et al., the deformation of material can be achieved by two aspects: water absorption and dehydration [60]. An example that exhibited both absorption and dehydration properties is hydroxybutyl methacrylated CS, a modified CS polymer that is reversible, photocrosslinkable, and temperature-sensitive. It is reported that hydroxybutyl methacrylated CS was utilized as the bio-ink of 4D printing because of its self-modified ability in response to the temperature change. A lower temperature increases the water absorption, resulting in elongated pores and cracks and deformed bending, while a higher temperature dehydrates the construct so that it can be shrunken and reform the original shape [61]. Lithographic printing instruments, such as SLA, was a frequently used technique that mimics the complex biological structure [62].

However, hydroxybutyl methacrylate is restricted use in contact with skin, which should not be used in food-grade products, but this research gave an insight to discovering edible materials with CS and SLA printing resin. In addition, CS is a natural pH-sensitive polymer that exhibits deformation properties as pH differs [63]. Therefore, it is also possible to construct a 4D printing object with pH-induced shape- and structure-changing properties in the future. By using SLA technology, a detailed internal structure can be fulfilled, and the antibacterial ability of CS is placed with great expectations, which accelerate the productions of artificial tissue and personalized meat. As abovementioned, the variable time or pH can be utilized as strategies for developing novel bio-inks based on CS.

### 2.5. Chitosan with Anthocyanin in 4D Printing

**Figure 4:**
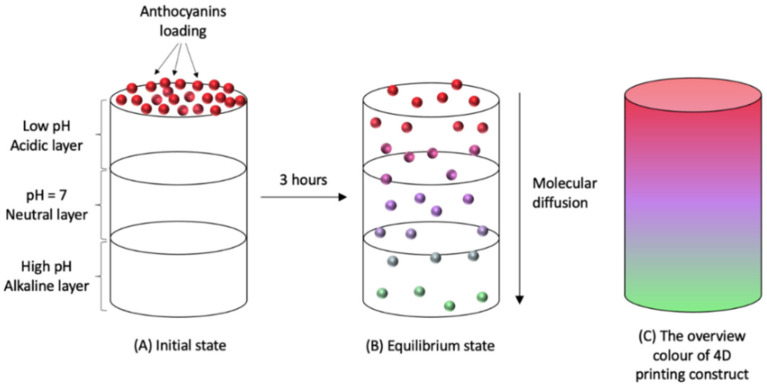
Time-dependent discoloring pattern of anthocyanin in different pH conditions; the scaffold of acidic, neutral, and alkaline layers is constructed by 3D printing. (**A**) Initial state when anthocyanins are just loaded on the top layer. (**B**) When the anthocyanin molecules reached equilibrium state after 3 h by molecular diffusion. (**C**) Overview color of 4D printing construct of anthocyanin.

Apart from deforming and reforming materials, other polymers that exhibit color-changing properties in response to stimuli also meet the definition of 4D printing material. Customers prefer to purchase food with fantastic colors, which proposes an excellent introduction of 4D printing research of color change in the food industry [60]. Hence, the achievement of the combination of CS and anthocyanin as multi-material in film production inspires the applications of CS and anthocyanin in the area of 4D printing. For instance, a research group combined the anthocyanin-potato starch layer on the top with the lemon juice layer on the bottom with pH difference, using a double-nozzle fused deposition model (FDM) printer. The pH gradient caused the color transformation of anthocyanin from reddish (low pH) to greenish (high pH), driven by gravitational molecular diffusion over time (Figure 4) [64]. Therefore, it is suggested to utilize edible anthocyanin as an autonomic discolor material induced by pH changes to achieve the fourth dimension of 3D printing [61].

Various studies have shown that products rich in anthocyanins enhance exercise recovery, proving that the antioxidant activity of anthocyanins is beneficial to human health [65]. Over the years, the discolor property of anthocyanin has been used in producing intelligent CS films that monitor pH changes. CS was less used in packaging films because of its low antioxidation, while the addition of anthocyanin significantly increases the antioxidation ability of CS film by intermolecular mutual effects, therefore making it an excellent substitute for plastic packages [41]. Anthocyanin–chitosan film was performed to detect the freshness and alert about a spoiled food product by changing color and has been applied in the packaging of milk [66], cream cheese [67], crucian fish [68], etc.

## 3. Whey Protein in 3D and 4D Printing

### 3.1. Characteristics of Whey Protein

Currently, there are four forms of WP product: WP concentrates (WPC), WP isolates (WPI), WP hydrolysate (WPH), and natural WP. These proteins are further processed to make dietary supplement powders and are facilitated in emulsion and gelation for many food products, such as ice cream and yogurt. More than that, the protein modification technologies also allow WP to participate in making edible films, hydrogels, and nanoparticles that are beneficial in various fields [69].

**Figure 5:**
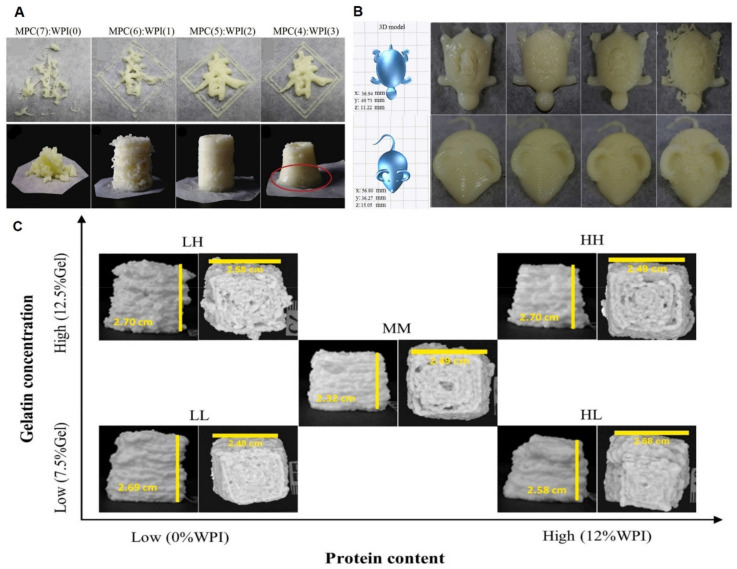
(**A**) 3D-printing constructs of different fractions of MPC and WPI [67]. (**B**) Different total protein content: a/a, 350 g/L; b/b, 400 g/L; c/c, 450 g/L; and d/d, 500 g/L milk protein gel preparation printing results. (**C**) View of 3D-printed gel morphology from the top and side: LL, 0% WPI–7.5% gelatine; LH, 0% WPI–12.5% gelatine; MM, 6% WPI–10% gelatine; HL, 12% WPI–7.5% gelatine; HH, 12% WPI–12.5% gelatine. Adapted from References [66,71,76], with permission from Elsevier.

WP, as an additive, was used to improve the stabilization and performance of 3D-printed constructs. WPs with polymeric and amphiphilic properties allowed them to firmly anchor to the oil–water interface [69]. Various research proved that the addition of WP can significantly enhance the printability and gel performance of the resulting bio-inks. Substituting a fraction of heat-induced WP isolate with microparticulate WP improved the stability by creating a viscous and elastic bio-ink. Liu et al. combined WP isolates and milk protein concentrate at a ratio of 2:5 to obtain the proper viscosity and strength for the best 3D printing performance, and the results played a significant role in promoting the development of high-protein food construction (Figure 5A) [70]. Moreover, Du’s research group found that adding WP to the konjac hybrid gel deformed the original structure and rebuilt a denser structure by crosslinking with the starch in konjac, which was non-conductive and had greater performance in FDM 3D printing [71].

Based on the potential properties of WP as an excellent bio-ink that significantly contributes to the printability and performance during the 3D printing process, modifications can also be applied to WP that allow for a range of adaptation [72]. Liu et al. used a WP isolate with 97.8% protein to emulsify the market-purchased soy oil and revealed a uniformly distributed gel-like emulsion after microfluidization, which greatly contributes to the stabilization of the emulsion. In addition, Liu et al. also tested the performance of WPI emulsion in 3D printing and concluded that the emulsion is suitable for the FDM technique and the modifiable solidification (gradually solidified as oil fraction increased) also left more availability for adjustment in 3D printing [73].

The performances and properties of five proteins and fruit/vegetable powder mixture including WP isolates (WPI) were evaluated as 3D printing bio-inks and concluded that the WPI with fruit/vegetable powders exhibited the lowest springiness and gumminess and highest color appearance and free water content [74]. Therefore, it is believed that the product of WPI with fruit/vegetable powders conforms more to children’s and elders’ dietary habits and needs. Hence, the future study can consider WPI as a bio-ink ingredient to construct 3D-printed edible training toys for children and snacks for the elderly. Liu et al. increased the viscosity and yield stress of the milk protein composite gel by increasing the total protein content (400–450 g/L) and improving the printability of the gel. However, the study also found that the printing quality was significantly reduced if the protein addition was too high (Figure 5 (B)) [75]. Riantiningtyas et al. combined gelatine in different concentration ratios with WPI to configure the ink. Due to the gel-softening effect of WPI, softer gels were produced as the WPI content increased, and it was observed that the samples became easier to extrude, while, at the same time, a decrease in the firmness and resilience of their products was observed. By blending gelatine and WPI to the right ratio, firm, stable, and well-extruded gels can be obtained (Figure 5C) [76].

### 3.2. Whey Protein in 3D-Printed Probiotic Encapsulation

Probiotics are microbials that support the main digestive function of intestines, increase the bioavailability of food intakes, and influence the mental condition and health [77]. However, probiotics are extremely sensitive to external environmental conditions, for instance, the low pH of gastric acid in the human body. Hence, the effectiveness of probiotics delivery in animals became a well-developed area of study worldwide. Recently, various research studies have applied 3D printing in the encapsulation of probiotics, using edible proteins or printable food materials that allowed the probiotics to survive under extreme conditions, for instance, 145 °C in baking [78] and 5 °C in the fridge and storage [79].

In 2019, the research conducted by Krunić et al. employed WP concentrates and WP hydrates with alginate in the protection and delivery of probiotics and retained 94% of the viability of probiotic cells effectively. The WP and alginate interaction exhibited higher porosity but stronger mechanical properties; in addition, the acid and bile tolerances of probiotics were enhanced compared to free cells [80]. Yoha et al. utilized fructooligosaccharides: WP: maltodextrin matrix, a high protein and fiber material, in the 3D printing to encapsulate probiotics. It is concluded that the survivability and viability of probiotics were almost retained by freeze-drying, spray-freeze-drying, and 3D-printing encapsulation [81]. Overall, the feasibility of encapsulating probiotics by using 3D printing of WP was further supported by various research studies [78,79,82,83,84].

### 3.3. Whey Protein in 4D Printing

Automatic changes in the chemical properties of a 3D-printing construct after applying an external stimulus are also considered as 4D printing, such as a change in chemical compositions. Phuhongsung et al. analyzed the rheological and chemical properties of a printed object consisting of soy protein isolates, carrageenan, and vanillin additive in 2020. Surprisingly they found that four flavor compounds were newly synthesized after microwaving the 3D-printed construct, and the fragrance intensity might be sensitive to the microwave temperature (higher temperature led to the stronger aroma). The experiment not only proposed the optimal formulation of soy protein isolates, carrageenan, and flavor additive but also confirmed the efficacy of 4D printing materials and their properties [85]. Both soy protein and WP are commonly used food-based proteins that have great contributions in formulating 3D printing inks [86]. However, the addition of dithiothreitol that improves the printing performance of soy protein is toxic to cells by generating reactive oxygen species [87]; therefore, a food-grade ink ingredient should be considered as a substitution. As mentioned above, the mixture of WP isolates and other modified WPs meets the requirement of 3D printing; hence, it is suggested to replace soy protein isolates with WP isolates to study edible 4D-printed protein products.

It was well considered that bovine serum protein can be stimulated to common conditions, such as pH and ionic strength, and thus undergo reversible deformation, while employing enzymatic stimulation of protein hydrogels to trigger irreversible deformation and thus achieve a multi-stimulus response [88]. This innovative initiative introduces that protein-based hydrogels as 4D printing materials show great potential for future research in the direction of smart biomedical materials. WP, used as a protein matrix as the bovine serum protein, can also be considered as alternative material for future 4D printing.

## 4. The Advantages of Combing Chitosan and Whey Protein

Overall, CS and WP have been proved to have multiple advantages in improving the performance of 3D printing and the quality of products in the medicine, space, engineering, and food sectors. Chitosan-based hydrogel has excellent biocompatibility, biodegradability, and antibacterial activity; high water content; and low polymer content. It was used mainly in constructing the scaffold for the cell growth of animal tissues and organs [25]. WP derivatives (WPC, WPI, WPH, etc.) were used as additives to improve the printability, stability, and performance of bio-inks for the 3D printing of food products. In the last decade, scientists studied the molecular interactions between proteins and polysaccharides under different conditions, for instance, pH, concentration, and temperature. In the case of WP and CS, the formation of CS/WP requires greater pH values; this is because that they have little attraction when the pH is less than 4.5 and form a CS/WP complex through electrostatic attraction when the pH is greater than 5.3 [89,90]. As the addition of CS increases from 1:5 (CS/WP), the viscosity of CS/WP solution increases [90]. Therefore, the mixture of CS and WP forms a homogeneous solution with CS/WP complexes that inherits the chemical properties and nutritive values from both materials and makes it an excellent bio-ink for the 3D printing of food products.

CS, and WP were also widely utilized in 3D-printed packaging films, not only in food printing [40,47,91]. In order to overcome the limitation of the high-humidity sensitivity of CS, hydrophobic substances such as WP can be mixed with a CS matrix to prepare composite materials, thus improving the mechanical properties of CS film, making it more suitable for 3D printing [40]. WP was also used as a plasticizer in previous studies [45]. As above, it can be considered that WP and CS, the multifunctional natural biodegradable substances, performed broad development prospects in food packaging. The previous section mentioned that the structure of the CS film containing nanoparticles produced better antimicrobial properties and transfer efficiency. Similar microstructures were consistent with previous studies about CS–WP films [45], suggesting the possibility of combining 3D printing, nanoparticles, and CS–WP films in producing for food packaging, as a green substitution of plastic products.

When considering the 3D printing of cultured meat scaffolds, protein is necessary nutrition that supports the growth of muscle cells in vitro. There are many types of protein that have been proved to have beneficial in the 3D printing process, such as pectin, egg-white protein, WP isolate, etc. It is suggested for future studies to attempt combining CS scaffold with dairy additive WP, using multilayer printing strategies, which create a scaffold with controlled releasing of nutrition.

Processing of probiotics using WP, CS, and CS–WP as materials is feasible by using 3D printing technology. It was found that the non-digestive polysaccharide, CS, can be utilized as encapsulation material because it stabilizes the alginate [82]. Krunić et al. reported that the addition of CS to bio-ink significantly improved the protection and survival of probiotic bacteria [80]. As a potential prebiotic material, CS shares positive effects on the probiotic’s growth and gut health [83,84]. Therefore, future research can be focused on the composition of WP, CS, and alginate in 3D printing of better probiotic encapsulation.

Microgels made by a CS or WP also act as the stabilizer of high internal-phase emulsions (HIPEs). HIPEs is the advanced emulsion technique that increases the fraction (above 74%) of internal-phase material in the emulsion. The structure of emulsion is similar to foam that contains a low amount of liquid; therefore, many emulsion solutions are unstable regarding thermodynamical and kinetical properties [92]. Guo et al. [93] stated that the rigid conformation formed by protein and polysaccharide microgels showed stronger ability with stabilization of interfaces of emulsion. Another advantage of using HIPEs in food-grade industries is that HIPEs avoid using partially hydrogenated oils (PHOs) in the products, as they contain health-concerned trans-fatty acids [73]. In addition, the higher concentration of internal phase materials provided the emulsion with stable, precise properties, and high viscosity in 3D printing. Hence, HIPEs stabilized by CS or WP microgels are considered the ink of 3D printing, with excellent performance and printability, that can be utilized to make 3D-printed food products in the food-grade industry.

As mentioned above, the applications of HIPEs were found mainly in areas such as packaging materials, food 3D printing, cosmetics, and others. Certain solid particles can bind to the surface of the emulsion droplets, and the resulting gel-like solution is called pickering emulsion. The combinations of pickering emulsions, with long shelf life and protection of sensitive compounds, and rapid prototyping 3D printing technologies are of increasing interest to researchers. As concluded by Liu et al., soybean proteins, zein, milk proteins (including WP), starch, and CS are effective solid emulsifiers that modify the liquid oil into gel-like and printable materials in the form of pickering emulsions [73,94]. Shahbazi et al. used modified microcrystalline cellulose to replace the grease to form a pickering emulsion that improved the ink’s printing performance and mechanical strength and reduced the co-efficient of friction, thus resulting in a smoother texture, while improving the rough appearance of the 3D-printed product and increasing stability [95]. In the future, the technology could be applied to make solid fat substitutes in the search for more personalized products.

Liu et al. performed research on the pickering emulsion prepared by WP isolates by using microfluidization and examined its rheological properties, gel strength, microscopic structures, and 3D-printing performances. The emulsion with increasing oil fraction showed very stable structural performances, due to stronger protein–protein interactions and smaller droplets caused by microfluidization, which allowed 60 days of storage. This finding suggested that microfluidization provided a great improvement in emulsion stability, viscosity, shear recovery, gel strength, and anti-freezing ability [96], which were consistent with their preliminary studies [97]. Higher oil fraction (0.4-0.6) resulted in higher protein aggregation; thus, the result was a more rigid structure that can be utilized in 3D printing. However, the printed products of high oil fraction of 0.6 had an unsmooth surface, which might be caused by a high impulsive force when processing in the microfluidizer [73].

All in all, HIPEs modified by WP isolate have good stability during storage and 3D printing by adjusting the oil content and exhibit various structural properties. It is recommended that further studies focus on HIPE supported by WP as a substitute for unhealthy partially hydrogenated oils and develop other 3D printing products in the food-grade field. Based on this, similar studies utilizing CS as an emulsifier of HIPEs are encouraged. Moreover, the combination of CS and WP provides better printability and stability; it is suggested to discover the rheology and other features of CS/WP-combined HIPEs in 3D and 4D printing.

To better understand the full article, we have summarized and compared the application of CS, WP and CS–WP with other different additives in 3D/4D printing and the characteristics of the printed end product (Table 3). As shown, the use of inks with CS and WP configurations for 3D printing and future 4D printing technology is a promising direction for future food industry. It is expected to point the way for future research and development of CS and WP, as well as better economic benefits.

## 5. Conclusions, Challenges, and Perspectives

In this review, the relationship between CS and WP in 3D and 4D printing for the food industry was discussed. When combined with CS and WP, HIPEs are produced with better stability, 3D printing capabilities, and possibly 4D printing properties in the future. HIPEs are mainly suitable for health and economic products, such as packaging materials, food 3D printing, and cosmetics. A vast majority of 3D-printed food products require post-processing, and post-processing methods to ensure that the appearance, quality, and taste of food products are one of the future research directions to produce products that better meet the specific needs of consumers. This paper provides the status of the application of CS, as well as WP in 3D printing technology, which provides some reference for the printing of other materials and provides new ideas to improve the feasibility of customized food production and development. In terms of 4D printing, recent studies have shown great potential in meeting customized and specific requirements of the food-grade industry, such as shape variation, color variation, and androgen variation.

As a continuation of this topic, many efforts have been made to add some specific composite materials in food printing to achieve changes in the space and time dimensions, for the nutritional value of the added materials, as well as stability, including consumer satisfaction, is the direction of future 4D printing needs to be studied. In the field of food 4D printing, spontaneous color change or deformation can be achieved by some post-processing (e.g., magnetic-field interference), and it is a great challenge to ensure the stability of the material and the sensory quality of the product is rarely affected in the post-processing. At the same time, more suitable post-processing methods can be developed to improve efficiency and even achieve color change and deformation of printed food simultaneously. In addition, from an environmental point of view, environmental friendliness or biodegradability limits the application in the biomedical field. Meanwhile, consumer acceptance is an important factor that cannot be ignored, and further market research should be conducted to cater to consumer preferences. As mentioned above, more efforts should be performed and explored for the application of CS and WP in food printing.

Future research scholars can focus on investigating the relationship between the internal structure of food products and textural properties, such as porosity, to establish numerical models to provide data support for 3D printing of custom-designed food products. Importantly, food printing needs to be further explored and developed in terms of delivery of specific nutrients or functional ingredients, fat substitutes, meat substitutes, 4D printing, and unexplored instruments or mathematical models. More research is needed for printed foods to meet the health needs and nutritional control of people of different occupations, ages, and lifestyles and to demonstrate greater commercial value.

## Figures and Tables

**Table 1:** Summary of materials, benefits, and drawbacks of 3D and 4D printing techniques. Adapted from References [8,9], with permission from Elsevier.

Methods		Materials	Benefits	Drawbacks	Related Equipment	Applications
3D printing techniques	Fused deposition modeling (FDM)	Blended polymer filament, pure polymer filament, and thermoplastic polymers	Cheap material, efficient, and simple manipulation	Poor mechanical strength, material is limited to thermoplastic, and layer-by-layer control [8,9]	Equipment, extrusion, and selective laser sintering	Engineering, customized products, medical, aerospace, and others.
	Stereolithography (SLA)	Ceramic monomers, polymers or photopolymers, and composites [10]	High quality, fine resolution, and fabrication accuracy	Time-consuming and expensive, material is limited to large volume production		
	Electro-hydraulic printing (EHDP)	Insulating and conducting polymers; suspension of nanotubes and nanoparticles [11]	High resolution, low cost, fine resolution, timesaving, and free design [12]	Material is limited to large-scale object		
	Powder bed fusion (SLS, SLM)	Compressed metal, alloys, polymers, and ceramic powders	High definition and quality	Slow printingExpensiveHigh porosity in the binder method [8,9]		
4D printing technique		Smart materials, depending on the type of stimulus received, are classified as physical, chemical and biologically responsive stimulus materials [13]	It can undergo reversible or irreversible functional changes, high resolution, lowering the transformation temperature Tg, the continuous shape change, can print complex structures [14]	Still in its infancy, the need for repeated responses can damage the mechanical properties of the structure; most materials can only respond to one stimulus [13]	Improved nozzles, binders and selective lasers	Smart medical devices. cell/drug or protein carriers, drug delivery, intelligent bio-robotics, furniture, construction, and others.

**Table 2:** Benefits and drawbacks of in applied 3D/4D printing.

Categories	Benefits	Drawbacks
3D printing	Easy to operate, low-cost, and efficient; can produce customized food products to meet consumer needs and can also be bioprinted to culture functional tissue structures and organs. [13]. This technology will enable the use of no additives in food [17].	Only the original state of the printed object is considered, failing to take into account the life of the creature and its dynamic nature [13]. Low consumer acceptance of food produced through 3D printing.
4D printing	Capable of creating complex structures with high precision and continuous reversible or irreversible morphological changes by responding to stimuli [14]. Bioprinted products are cytocompatible.	In the biomedical field, the existing technology to achieve morphological changes and operational precision still needs to be improved, while the stimulus to trigger the response should be found in a gentler way for application to living organisms. In the food sector, the technology is costly, most research areas are narrow, and the efficiency of the transformation needs to be improved [4].

**Table 3:** Applications of CS, WP, and CS–WP with other different additives in 3D/4D printing and the characteristics of the printed products.

Categories	Applications	Characteristics of the Products
CS	Food packaging film [46]	Good antibacterial properties, biodegradable and non-toxic for fresh food storage
	Cell scaffold [52]	Edible, heat resistant, antibacterial and biocompatible
	Hydrogel [46]	Good anti-bacterial properties
WP	Edible films, hydrogels, and nanoparticles [69]	Improved stability
CS–WP	Food printing [90]	Inherits the chemical properties and nutritional value of both materials
	3D-printed packaging film [40,47,91]	Overcomes the high moisture sensitivity of CS, improves the mechanical properties of the film, and promises to be a green alternative to plastic products
	Scaffolding that could be applied to cultured meat in the future	It should be possible to achieve controlled nutrient release
	Processed probiotics [82,83,84]	Significantly improves the survival rate of probiotics and facilitates probiotic growth for intestinal health
	The prepared microgels can be used as stabilizers for HIPEs [92]	Improves stability and is expected to replace PHO
	Pickering emulsions [95]	Smoother texture, improving the rough appearance of 3D-printed products and increasing stability
CS–protein	Food packaging film [42]	higher flexibility and elongation property
Film with CS nanoparticles	Food packaging film [46]	Better stability; lower water vapor permeability and solubility
CS/HNTs–TP composite material	Food packaging film [47]	Significant increase in fresh fruit freshness
Encapsulating proteins in CS nanogels and incorporating them into mixed suspensions	Cultured meat [48]	Enables sustained release of macromolecular proteins to guide tissue growth, with good biocompatibility
CMC–CS	Cultured meat [52]	Increases the total porosity of the film for cell growth, while enhancing the mechanical properties of the scaffold and improving degradability
CS–carrageenan composite gel	Food packaging film [57]	Reduces oxidation and discoloration, prolongs the shelf life of food and retards the growth of microorganisms
Nanocoated polyelectrolyte gels of CS and pectin	Food packaging film [59]	Ability to keep fruits fresh
Hydroxybutyl methacrylate CS	4D printing [61]	Possibility to construct a 4D-printed object with pH-induced shape and structure change properties; detailed internal structures can be achieved by using SLA technology
CS–anthocyanins	4D printing packaging film [41]	Detects freshness and alerts to spoilage by changing color, with improved antioxidant capacity
WP–konjac blend gel	FDM printing [71]	Non-conductive and more dense structure for improved stability
Fruit/vegetable powder mixture including WP isolates (WPIs)	3D-printed edible training toys for children and snacks for the elderly [74]	the lowest springiness and gumminess and highest color appearance and free water content
WPI–gelatin	3D-printed yoghurt-based food [76]	Stronger and more stable end-products printed with the right proportion of WPI-gelatin
WP–MPC	Print foods with a high protein structure [67]	Better stability
WP–sodium alginate	Protection and delivery of probiotics [80]	Higher porosity for better mechanical properties
Fructooligosaccharides: WP: maltodextrin matrix	Encapsulated Probiotics [81]	Survival and viability of probiotics are preserved
Soy protein isolate, carrageenan and vanillin	4D printing [85]	New fragrance compounds synthesized
Bovine-serum-protein-based protein gel	4D printing material for future smart biomedical applications [88]	Responds to stimuli such as pH, ionic strength, etc., and can also be subjected to enzymatic stimuli that trigger irreversible deformations

## Data Availability

Not applicable.
